# Popliteal artery injury associated with multiligament knee injuries and tibial plateau fractures: A 20‐year retrospective cohort study

**DOI:** 10.1002/jeo2.70943

**Published:** 2026-10-05

**Authors:** Efstathios Konstantinou, Nikolaos Stefanou, Christina Arnaoutoglou, Zoe Dailiana, Sokratis Varitimidis, Michael Hantes

**Affiliations:** ^1^ Department of Orthopaedic Surgery and Musculoskeletal Trauma, University Hospital of Larissa, School of Health Sciences University of Thessaly Larissa Greece

**Keywords:** functional outcomes, limb salvage, multiligament knee injury, popliteal artery injury, tibial plateau fracture

## Abstract

**Purpose:**

Popliteal artery injury (PAI) around the knee is a limb‐threatening condition typically associated with high‐energy trauma, particularly multiligament knee injuries (MLKIs) and tibial plateau fractures. This study aimed to analyse injury patterns, management, complications and functional outcomes, with the hypothesis that fracture‐related PAI would be associated with better functional outcomes than MLKI‐associated PAI.

**Methods:**

A retrospective review was conducted of patients treated for PAI at a tertiary referral centre from 2003 to 2023. Patients were eligible if computed tomography angiography confirmed PAI associated with MLKI or a high‐energy tibial plateau fracture. Demographics, injury characteristics, surgical management and complications were recorded. Functional outcomes were assessed using the International Knee Documentation Committee (IKDC) score and visual analogue scale (VAS) for pain.

**Results:**

Forty‐eight patients (mean age, 44.1 years; 68.7% male) were included: 36 with MLKIs and 12 with fracture‐related vascular injuries. Vascular procedures comprised popliteal bypass grafting in 30 patients (62.5%), venous patch angioplasty in 10 (20.8%) and primary arterial repair in 8 (16.7%). External fixation was applied to all patients. Peroneal nerve injury occurred in 15 patients (31.3%). Amputation was required in five patients (10.4%), all within the MLKI group and was associated with delayed presentation, advanced soft‐tissue compromise and neurovascular injury severity at the time of referral. At final follow‐up, the fracture group had higher IKDC scores (85.4 vs. 74.2; *p* = 0.032) and lower VAS pain scores (2.39 vs. 3.47; *p* = 0.049) than the MLKI group.

**Conclusion:**

In this cohort, fracture‐related PAI was associated with better functional outcomes than MLKI‐associated PAI, whereas all amputations occurred in the MLKI group after delayed presentation and referral. These findings emphasize the importance of timely diagnosis, referral and coordinated vascular–orthopaedic management.

**Level of Evidence:**

Level III, retrospective comparative study.

**Abbreviations**
ABI: ankle–brachial index
CI: confidence interval
CT: computed tomography
IKDC: International Knee Documentation Committee
KD: knee dislocation
MLKI: multiligament knee injury
PAI: popliteal artery injury
PCL: posterior cruciate ligament
VAS: visual analogue scale

## INTRODUCTION

Popliteal artery injury (PAI) is a rare but limb‐threatening complication of trauma around the knee [5, 14]. It is most commonly associated with high‐energy tibiofemoral dislocations and periarticular fractures, particularly tibial plateau fractures [2, 4], although it may also occur after low‐energy knee dislocation (KD) [2]. In a nationwide cohort, PAI occurred in 3.4% of patients with KD or multiligament knee injury (MLKI), increasing to 8.2% when only tibiofemoral dislocations were considered, compared with 0.2% of periarticular fractures [2]. Reported rates vary according to cohort definition and may reach 18% in series of operatively treated MLKIs [15].

Careful clinical vascular examination is the first step in suspected PAI, with absent or diminished distal pulses, distal ischaemia, an expanding haematoma, bruit, neurological deficit or severe swelling warranting urgent investigation [8, 12]. Current guidelines recommend immediate computed tomography angiography (CTA) when significant vascular injury cannot be excluded clinically [22]. Peroneal nerve injury is an important warning sign of associated PAI [11, 14], while ischaemia exceeding 6–8 h markedly increases the risk of amputation [8, 10, 23].

However, comparative data on management and outcomes between these injury patterns remain limited. The primary objective of this study was to retrospectively analyse the clinical characteristics, management and outcomes of patients with PAI associated with traumatic MLKIs and high‐energy tibial plateau fractures. Secondary objectives included evaluation of vascular procedures, complications, fracture union and functional outcomes, as well as an exploratory comparison between MLKI‐ and fracture‐related vascular injuries. MLKIs are frequently associated with extensive soft‐tissue disruption, persistent ligamentous instability and concomitant neurovascular injury, which may adversely affect functional recovery [7, 15, 20]. It was therefore hypothesized that fracture‐related PAI would be associated with better functional outcomes than MLKI‐associated PAI.

## MATERIAL AND METHODS

This retrospective study included all patients treated for PAI at a single tertiary orthopaedic centre between January 2003 and December 2023. Our centre serves as a regional referral hospital for complex limb trauma and revascularization procedures. Institutional review board approval was obtained prior to study initiation (approval number: 48189/07‐11‐25). This study represents Level III evidence as a retrospective comparative study.

Potential cases were identified from the electronic operating theatre database using combined keyword searches. Terms related to vascular injury or intervention—including ‘popliteal artery’, ‘popliteal artery injury’, ‘vessel injury’, ‘vascular injury’, ‘arterial injury’, ‘vascular repair’, ‘arterial repair’, ‘vascular intervention’, ‘revascularization’ and ‘Gustilo–Anderson Type IIIC’—were combined with injury‐related terms including ‘knee dislocation’, ‘multiligament knee injury’, ‘tibial plateau fracture’ and ‘proximal tibial fracture’. Variations of these terms were also used to account for differences in terminology across the study period. The retrieved records were then reviewed against the eligibility criteria. Only records retrieved through the combined keyword strategy were assessed, as searches using isolated diagnostic terms produced nonspecific results. Eligibility was assessed by the first two authors, both orthopaedic surgeons. Discrepancies were resolved through discussion between them; when uncertainty remained, a senior author with detailed knowledge of the cohort was consulted. MLKI was defined as injury involving at least two major knee ligament complexes and was classified according to the Schenck system [20]. Inclusion criteria were radiologically confirmed PAI in the context of a MLKI consistent with traumatic KD or a high‐energy tibial plateau fracture and surgical treatment at our institution during the defined period.

Patients were excluded if PAI occurred distal to the trifurcation. Additionally, cases involving isolated vascular injuries without associated osseous or ligamentous trauma around the knee were also excluded to maintain focus on musculoskeletal‐associated vascular injuries, as defined in prior studies [2, 5, 13]. Patients with iatrogenic PAI related to total knee arthroplasty, osteotomy, arthroscopy, ligament reconstruction or other surgical procedures around the knee were excluded, as were patients with PAI associated with prosthetic instability or dislocation.

In patients with suspected vascular injury, PAI was confirmed by CTA in all cases, and all examinations were reviewed and formally reported by a radiologist. The decision to perform vascular imaging was based on the presence of hard or soft signs during patient evaluation. Hard signs comprised active pulsatile or massive bleeding, a rapidly expanding or pulsatile haematoma, bruit or thrill and clinical evidence of arterial insufficiency or ischaemia. Soft signs included a history of arterial bleeding, a diminished unilateral distal pulse, a small non‐pulsatile haematoma over a named artery, proximity of the injury to a named artery or a neurological deficit involving an adjacent nerve [21]. Initial vascular assessment included clinical evaluation of distal perfusion and pulse examination, with Doppler ultrasonography performed by the vascular surgery team when vascular compromise was suspected, prior to definitive imaging. Ankle–brachial index (ABI) was not routinely used as a primary screening tool in the acute trauma setting. In patients with high clinical suspicion of vascular injury, CT angiography was performed directly to expedite diagnosis and surgical planning. ABI was used selectively in equivocal or delayed presentations but was not systematically obtained prior to CT angiography. CT angiography provided both high sensitivity and rapid assessment of vessel integrity and was standard practice throughout the study period [21, 22]. Conventional angiography was performed selectively, mostly before 2010, when multidetector CT was not consistently available [3, 14].

Data extracted included age, sex, injury mechanism, presence of peroneal nerve injury, vascular procedure type, fixation method and soft‐tissue complications. The treating orthopaedic surgeon classified each MLKI according to the Schenck system [20]. Follow‐up data were obtained from outpatient records or structured telephone interviews when clinic documentation was limited.

Peroneal nerve injury was diagnosed clinically by the treating team and identified retrospectively from neurological findings documented in the medical records. Documentation included motor and/or sensory deficits consistent with common peroneal nerve dysfunction. A standardized neurological grading system was not consistently applied throughout the study period.

Functional outcomes were assessed using the International Knee Documentation Committee (IKDC) subjective score [9] and visual analogue scale (VAS) for pain. These were collected either during routine follow‐up or retrospectively via telephone, when in‐person evaluation was not available. For the tibial plateau fracture subgroup, fracture union and nonunion were recorded, and fractures were classified using the Schatzker system [19]. Fracture healing was assessed using serial radiographs and clinical examination, including progression of callus formation, maintenance of alignment and pain‐free progression to weight‐bearing. CT was performed selectively when nonunion was suspected due to persistent symptoms or insufficient radiographic evidence of healing. Although the Rasmussen score represents a fracture‐specific outcome tool for tibial plateau fractures [18], it was not consistently available retrospectively across the 20‐year study period and could therefore not be reliably analysed. In addition, it cannot be applied to MLKIs and therefore would not allow uniform comparison across the heterogeneous cohort. IKDC was selected as the common functional measure because it can be applied to both fractures and MLKIs, despite being less specific for fractures. Similarly, Mangled Extremity Severity Score (MESS) scores were not included, as several variables required for reliable retrospective calculation were not consistently documented throughout the 20‐year study period.

The sequence of skeletal stabilization and revascularization was individualized according to limb viability, fracture configuration, soft‐tissue injury and vascular surgery requirements. In most cases, provisional external fixation was initially applied in a configuration facilitating vascular access and revascularization. Following vascular repair, the external fixator was revised or repositioned when necessary to provide more definitive stabilization and protect the vascular reconstruction.

Bypass grafting was primarily used in cases of segmental arterial loss, thrombosis or extensive vessel damage precluding direct repair. Venous patch angioplasty was performed in partial arterial injuries with preserved vessel continuity, whereas primary repair was reserved for limited focal injuries permitting tension‐free reconstruction. Fasciotomy was typically performed during the initial revascularization procedure in patients with prolonged ischaemia, severe soft‐tissue swelling, high‐energy crush injury, delayed presentation or clinical concern for compartment syndrome.

Multiligament knee reconstructions were carried out in a staged manner after revascularization, with timing ranging from 3 to 6 months depending on vascular stability, soft‐tissue conditions and knee motion, using autografts and allografts in all reconstructions, with Achilles tendon allograft used mainly for posterior cruciate ligament (PCL) reconstructions when required. Reconstruction most commonly involved the central ligaments, with collateral ligament repair or reconstruction performed when residual instability was present, while posterolateral or posteromedial corner injuries were repaired or reconstructed in the presence of persistent coronal or rotational instability after assessment of the central ligament injuries.

Descriptive statistics were applied. Continuous variables are reported as means with standard deviations. Categorical variables are shown as absolute numbers and percentages. Exploratory comparisons between patients with MLKIs and those with fracture‐related vascular injuries were performed using independent *t* tests for continuous outcomes (age, IKDC and VAS scores) and Fisher's exact test for categorical variables (sex, reoperations, infections, amputations and peroneal nerve injury). Statistical significance was set at *p* < 0.05.

## RESULTS

A total of 48 patients with PAI were included. Demographic characteristics according to injury pattern are summarized in Table 1. There were no statistically significant differences between the groups in age or sex distribution.

**Table 1:** Demographic characteristics.

Demographic characteristic	Overall cohort (*N* = 48)	MLKI group (*n* = 36)	Fracture group (*n* = 12)	*p* value
Age, years, mean ± SD	44.1 ± 10.0	44.6 ± 9.6	42.6 ± 11.0	0.549
Male sex, *n* (%)	33 (68.7)	24 (66.7)	9 (75.0)	0.728

*Note*: Values are presented as mean ± standard deviation or number (%).

Abbreviations: MLKI, multiligament knee injury; SD, standard deviation.

Thirty‐six patients sustained MLKIs consistent with KD, while 12 patients sustained fracture‐related vascular injuries. Injury mechanisms and injury classifications are summarized in Table 2. All four open fractures (33.3% of the fracture group) required arterial repair and were therefore classified as Gustilo–Anderson Type IIIC [21].

**Table 2:** Injury mechanisms and classifications within the study cohort.

Injury characteristic	*n* (%)
Injury mechanism (overall cohort, *N* = 48)	
Motor vehicle accident	18 (37.5)
Fall from height	11 (22.9)
Work‐related trauma	10 (20.8)
Sports injury	9 (18.7)
Schenck classification (MLKI subgroup)	36 (75.0%)
KDII	1 (2.8)
KDIIIM	18 (50.0)
KDIIIL	10 (27.8)
KDIV	7 (19.4)
Schatzker classification (fracture subgroup)	12 (25.0%)
Type IV	5 (41.7)
Type V	1 (8.3)
Type VI	6 (50.0)

*Note*: Values are presented as number (%).

Abbreviation: MLKI, multiligament knee injury.

Surgical procedures, vascular reconstructions and relevant treatment intervals are summarized in Table 3.

**Table 3:** Surgical procedures, vascular reconstructions and treatment intervals in the study cohort.

Operative management	Overall cohort (*N* = 48)
External fixation, *n* (%)	48 (100.0)
Definitive plate osteosynthesis, *n*/*N* (%)^a^	10/12 (83.3)
Mean time to definitive plate osteosynthesis, days	12
Popliteal bypass, *n* (%)	30 (62.5)
Venous patch angioplasty, *n* (%)	10 (20.8)
Primary arterial repair, *n* (%)	8 (16.7)
Fasciotomy, *n* (%)	29 (60.4)
Time from injury to restoration of arterial flow, mean ± SD, hours	7.0 ± 2.1

*Note*: Percentages are based on the overall cohort (*N* = 48), unless otherwise indicated.

Abbreviation: SD, standard deviation.

^a^ Calculated within the fracture subgroup (*n* = 12).

Two patients in the MLKI group had delayed vascular presentation. In both cases, ischaemic signs were documented 2–3 days after the initial injury despite initially detectable peripheral pulses and no documented early signs of vascular compromise.

Among the 12 fracture cases, 10 (83.3%) achieved union, whereas 2 (16.7%) developed nonunion and required revision surgery with bone grafting; both subsequently achieved union.

At a mean final follow‐up of 6.4 ± 2.5 years, patients in the fracture group had significantly higher IKDC scores (*p* = 0.032) and lower VAS pain scores (*p* = 0.049) than those in the MLKI group (Table 4).

**Table 4:** Complications and functional outcomes according to injury pattern.

Outcome	MLKI group (*n* = 36)	Fracture group (*n* = 12)	*p* value
IKDC score, mean ± SD	74.2 ± 17.3	85.4 ± 15.1	0.032
VAS pain score, mean ± SD	3.47 ± 1.43	2.39 ± 1.71	0.049
Reoperations, *n* (%)	15 (41.7%)	4 (33.3%)	0.739
Infections, *n* (%)	4 (11.1%)	1 (8.3%)	1.000
Amputations, *n* (%)	5 (13.9%)	0 (0.0%)	0.312
Peroneal nerve injury, *n* (%)	13 (36.1%)	2 (16.7%)	0.292

*Note*: Values are presented as mean ± SD or number (%).

Abbreviations: IKDC, International Knee Documentation Committee; MLKI, multiligament knee injury; SD, standard deviation; VAS, visual analogue scale.

Five infections (10.4%) were recorded, including one deep infection in the fracture subgroup. Five patients (10.4%) underwent amputation, all in the MLKI group; no amputations occurred in the fracture group. All amputations involved patients referred from facilities without on‐site vascular surgery after either no vascular intervention or temporary external fixation alone. At referral, these patients had prolonged ischaemia and advanced soft‐tissue compromise.

Peroneal nerve injury was identified in 15 patients (31.3%). Most deficits were documented during the initial emergency department assessment. At final follow‐up, most affected patients demonstrated complete or partial neurological recovery; persistent deficits mainly comprised dorsiflexion weakness, sensory disturbance in the common peroneal nerve distribution or both.

There were no statistically significant between‐group differences in reoperation, infection or amputation rates. Detailed comparisons of functional outcomes and complications according to injury pattern are presented in Table 4.

## DISCUSSION

The present study evaluated popliteal artery injuries in the setting of MLKIs and tibial plateau fractures in a tertiary referral centre. The main findings were that MLKIs accounted for the majority of cases within this PAI cohort, whereas fracture‐related vascular injuries were associated with better functional outcomes, with higher IKDC scores and lower VAS pain scores at final follow‐up. However, the incidence of PAI among all patients with MLKI or tibial plateau fracture was not assessed. Amputation occurred exclusively in the MLKI group and was consistently observed in patients with delayed presentation following initial management at outside institutions without immediate vascular surgery support. Peroneal nerve injury was present in nearly one‐third of patients, reflecting the severity of these injuries.

PAI is a limb‐threatening condition most frequently encountered after high‐energy trauma, including KDs and complex periarticular fractures, and has been consistently associated with high rates of complications and amputation in the absence of timely diagnosis and intervention [2, 13]. Early recognition, appropriate imaging and rapid revascularization remain critical determinants of limb salvage [3, 5, 14].

Mechanism of injury in this cohort primarily involved high‐energy trauma, with motor vehicle accidents and falls accounting for more than half of all cases. This pattern is consistent with prior series identifying traffic‐related trauma and falls as the dominant causes of vascular injury around the knee [2, 16, 20]. While less frequent, sports and work‐related trauma remain clinically significant contributors, particularly in younger or working‐age populations [1, 5].

In our cohort, MLKI represented the majority of cases, consistent with previous reports highlighting the vulnerability of the popliteal artery during KD [15, 20]. Fracture‐related vascular injuries were less common but uniformly involved high‐energy tibial plateau fractures, primarily Schatzker IV–VI, a pattern repeatedly associated with arterial compromise in the literature [11]. Recent work specifically evaluating tibial plateau fractures with arterial injury supports similar injury distributions and outcomes [4].

Peroneal nerve injury was present in nearly one‐third of our patients and, as previously reported as a major soft sign, serves as a valuable clinical indicator of associated vascular injury. Natsuhara et al. found an odds ratio of 20.6 for vascular injury in the presence of a peroneal nerve deficit [14], a finding confirmed in more recent trauma literature as well [15, 17]. This relationship reinforces the importance of careful neurological assessment in suspected MLKI or high‐energy tibial plateau fractures. In the present cohort, peroneal nerve injury was more frequently observed in the MLKI group, which also demonstrated inferior functional outcomes and accounted for all amputations. Although a direct causal relationship cannot be established from the current data, these findings support the concept that peroneal nerve injury may represent a marker of greater injury severity and soft‐tissue compromise in complex knee trauma.

The choice of imaging modality plays a pivotal role in timely diagnosis and limb salvage. In our series, CTA was performed in all included patients presenting acutely to confirm vascular injury and expedite surgical planning, reflecting the high‐energy nature of these injuries, the frequent presence of soft signs and the referral‐centre setting. This practice is supported by reports emphasizing CTA following careful clinical examination when vascular injury is suspected [3, 8, 13]. Clinical vascular examination remains the first step; however, palpable distal pulses do not reliably exclude significant arterial injury [12, 22]. Recent reviews of combined musculoskeletal and vascular trauma similarly emphasize the importance of early CTA in suspected PAI [21]. The 2025 guidelines recommend immediate CTA as the primary imaging modality when significant vascular injury cannot be excluded clinically, provided that imaging does not delay urgent treatment [22]. This is particularly relevant in KDs and high‐energy tibial plateau fractures, where vascular injury may be clinically occult and prompt imaging can assist preoperative planning [2, 4, 22].

Two patients with MLKI in our series exhibited delayed vascular presentation, with ischaemic symptoms emerging 48–72 h after the index injury despite initially detectable peripheral pulses. These cases underline that palpable distal pulses do not exclude PAI. Although most vascular injuries present acutely, similar cases of late‐onset arterial compromise have been described and are often attributed to evolving intimal injury or thrombosis, resulting in worse outcomes when diagnosis is delayed [10, 16]. Given the potentially devastating consequences of missed PAI, a low threshold for CTA is warranted in patients with MLKI even when the initial pulse examination is reassuring. In this setting, the potential benefit of detecting an occult vascular injury outweighs the limited risks of diagnostic CTA. Continued vigilance and repeated clinical assessment remain essential, with repeat imaging when new or unexpected symptoms develop [21].

The surgical approach to PAI varies depending on the mechanism, timing and extent of associated damage. In our cohort, popliteal bypass was the most commonly performed procedure, followed by venous patch repair and direct arterial repair. This distribution is consistent with previously reported series, in which bypass was often favoured due to segmental injury or thrombosis precluding primary repair [1, 5, 6]. A multidisciplinary approach is essential; orthopaedic stabilization and vascular repair must be coordinated to preserve limb viability. Several authors have emphasized that limb salvage is achievable even in complex cases with combined vascular and orthopaedic trauma, provided timely intervention and coordinated care are delivered [5, 6, 23].

Fasciotomy was performed in more than half of our cases, in line with the literature that supports its liberal use to prevent compartment syndrome, particularly in ischaemia‐reperfusion scenarios [5, 17]. The need for multiple surgeries was common, reflecting the complexity of these injuries and the staged approach often required for definitive reconstruction. Functional outcomes, as measured by IKDC and VAS, were moderate in our series, mirroring the long‐term morbidity seen in similar cohorts [1, 5]. In the present cohort, IKDC and VAS scores differed between MLKI‐associated and fracture‐related vascular injuries, with better outcomes observed in patients with fractures. This may reflect the more extensive capsuloligamentous and soft‐tissue disruption associated with MLKI, with residual instability, restricted motion and concomitant neurological impairment potentially contributing to poorer long‐term function [7, 15, 20]. Clinically, these findings may inform patient counselling regarding expected recovery and guide rehabilitation and follow‐up. Given the heterogeneity of injury patterns, limited sample size and unadjusted comparisons, these findings should be interpreted as exploratory rather than definitive. The Rasmussen score, although widely used for tibial plateau fractures [12, 18], could not be applied uniformly to our mixed cohort and was therefore not used; IKDC was selected as a common functional measure across both groups.

Time to revascularization remains one of the most critical predictors of limb salvage. Ischaemia extending beyond 6 and 8 h significantly increases the risk of irreversible damage and amputation [2, 22, 23]. In our series, all amputations occurred in patients initially treated at other facilities lacking on‐site vascular surgery services and were associated with delayed referral, prolonged ischaemia and advanced soft‐tissue compromise at the time of presentation. Our institution acts as a regional referral centre, and delays in transfer or missed diagnoses contributed to poor outcomes in these cases, underscoring the importance of early recognition, rapid referral and availability of vascular expertise. This pattern is consistent with other reports linking delayed or fragmented care to higher amputation rates in PAI [2, 23]. These findings further support the need for regional trauma pathways facilitating early vascular assessment and timely transfer of suspected PAI cases to specialized multidisciplinary centres.

## LIMITATIONS

This study is subject to the inherent limitations of a retrospective design, including reliance on the quality and completeness of clinical documentation and potential underreporting of minor vascular injuries or delayed complications. Because cases were identified through operating theatre records and complete historical emergency department records were unavailable, the total number of patients screened and the number excluded could not be determined; consequently, the incidence of PAI among all patients with MLKI or tibial plateau fracture treated during the study period could not be calculated. The heterogeneity of the cohort, which included both MLKIs and tibial plateau fractures, limits direct comparison between groups. The limited number of patients with peroneal nerve injury precluded detailed subgroup analysis of neurological recovery and its association with functional outcomes. The absence of standardized follow‐up protocols and missing functional outcome data in some patients may limit the generalizability of the long‐term findings. Smoking status and other vascular risk factors were not consistently documented and therefore could not be analysed in relation to revascularization outcomes. Additionally, the tertiary referral‐centre setting may have introduced selection bias toward more severe or complex cases, particularly those presenting late from peripheral institutions [2, 23]. Furthermore, no multivariate analysis was performed because of the limited sample size and number of outcome events, restricting the identification of independent predictors and reducing statistical power to detect between‐group differences. Given the multiple comparisons, borderline statistically significant findings should also be interpreted cautiously because of the possibility of Type I error. Despite these limitations, the data provide valuable insight into the multidisciplinary management of PAI. Future prospective studies should aim to establish uniform treatment algorithms and standardized outcome tracking for these high‐risk injuries. The present findings support the development of regionalized trauma networks and protocols to ensure prompt vascular evaluation, particularly for patients with MLKI or high‐energy tibial plateau fracture at risk of PAI, which may further improve limb salvage [22].

## CONCLUSION

PAI around the knee remains a limb‐threatening condition requiring urgent diagnosis and specialized vascular and orthopaedic management. In the present cohort, fracture‐related vascular injuries were associated with better functional outcomes than MLKIs, although these findings should be interpreted cautiously given the heterogeneity of injury patterns and the absence of adjusted analyses. Delayed presentation and delayed referral from non‐specialized centres were associated with poor outcomes, including limb loss. Early vascular assessment, careful reassessment in high‐energy knee trauma, and timely transfer to specialized multidisciplinary centres are essential to optimize limb salvage and functional recovery.

## AUTHOR CONTRIBUTIONS

All listed authors have made substantial contributions to this work. Literature search, study design and primary manuscript preparation were performed by Efstathios Konstantinou and Nikolaos Stefanou. Data collection, analysis and interpretation of the results were performed by Efstathios Konstantinou and Nikolaos Stefanou. Statistical analysis was performed by Efstathios Konstantinou. Final manuscript drafting and critical revision were performed by Christina Arnaoutoglou, Zoe Dailiana, Sokratis Varitimidis and Michael Hantes. All authors have read and approved the final version of the manuscript.

## FUNDING INFORMATION

The authors have no funding to report.

## CONFLICT OF INTEREST STATEMENT

The authors declare no conflicts of interest.

## ETHICS STATEMENT

Institutional review board approval was obtained prior to study initiation from the Institutional Review Board of University Hospital of Larissa, Greece (approval number: 48189/07‐11‐25). The study was conducted in accordance with the principles of the Declaration of Helsinki. Patient consent was waived due to the retrospective nature of the study.

## Data Availability

Anonymised data from the study are available upon reasonable request.
